# Human penile ossification: case report

**DOI:** 10.1590/S1516-31802007000200012

**Published:** 2007-03-04

**Authors:** Homero Oliveira de Arruda, Hudson de Lima, Valdemar Ortiz

**Keywords:** Penile induration, Calcinosis, Heterotopic ossification, Penile diseases, Penis, Induração peniana, Calcinose, Ossifi cação heterotópica, Doenças do pênis, Pênis

## Abstract

**CONTEXT:**

Ossification in the human penis is such a rare condition that only 34 histologically evident cases have previously been reported. Among several conditions that have been correlated with this problem the most frequent is Peyronie disease. In all these conditions, human penile ossification appears to be a metaplastic bone formation process.

**CASE REPORT:**

A 59-year-old white man presented with a one-year history of slight pain upon erection and during intercourse. He also complained of hard plaque near the base of the penis. One year earlier, he had sustained blunt trauma during intercourse. Examination of the penis revealed a fixed firm mass extending over the proximal third of the penile shaft, measuring 3.0 × 3.0 × 2.0 cm and involving the corporal sponge, without surface extension. There was no impotence or other relevant clinical finding. Radiography on the penis revealed irregular calcification in the same position as the palpable mass and in the septum of the proximal inner third of the penis. The importance of this report lies in the extent of the human penile ossification, as demonstrated by the radiological and histological confirmation.

## RESUMO

**CONTEXTO:**

Ossificação do pênis humano é uma condição rara e apenas 34 casos histologicamente evidentes foram relatados. Várias condições foram relacionadas com o problema e o mais freqüente foi a Doença de Peyronie. Em todas, as ossificações parecem ser resultado de um processo de metaplasia com formação óssea.

**RELATO DO CASO:**

Um homem de 59 anos branco apresentou-se com história de leve dor no pênis durante as ereções há um ano. Ele também referia a presença de uma placa dura na base do pênis, que começou a crescer após um trauma na relação sexual. O exame do pênis revelou tratar-se de uma massa fixa, firme, no terço proximal do pênis de aproximadamente 3.0 × 3.0 × 2.0 cm, sem extensão para a superfície. Não havia impotência ou outra alteração clínica relevante. A radiografi a do pênis identificou extensa área calcificada no corpo esponjoso e região do septo, em correspondência à massa palpada. A importância deste relato é a extensão da ossificação de acordo com a radiologia e confirmação histológica.

## INTRODUCTION

Ossification in the human penis is such a rare condition that only 34 histologically evident cases have previously been reported in the literature. Several conditions have been correlated with this problem and the most frequent is Peyronie disease.^1-4^

In the present report, a further case of human penile ossification is presented. The importance of this report lies in the extent of the penile ossification, as demonstrated by the radiological and histological confirmation. In addition, we have reviewed all the previously reported cases and offer some comments concerning etiology.

## CASE REPORT

A 59-year-old white man was referred with a one-year history of slight pain upon erection and during sexual intercourse. He also complained of hard plaque near the base of the penis. One year earlier, he had sustained blunt trauma during intercourse, after which he began to experience pain when the penis became turgid. There was no history of metabolic disorder or erectile impotency.

**Figure 1:**
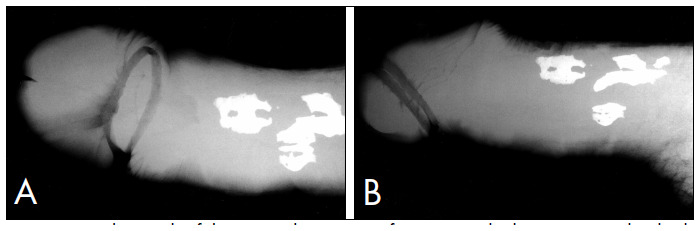
Radiograph of the penis showing ossification inside the septum and in both corpora cavernosa. A: frontal view; B: sagittal view.

**Figure 2:**
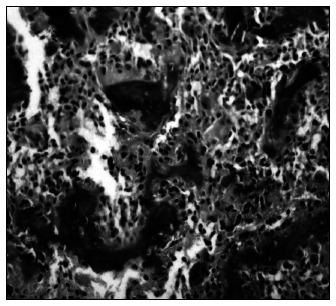
Photomicrograph of histological section from the lesion, showing metaplasia of bone tissue in the corpus spongiosum.

Examination of the penis revealed the presence of a firm fixed mass extending over the proximal third of the penile shaft. It was irregular, measuring 3.0 × 3.0 × 2.0 cm, and involved the corporal sponge without surface extension. There were no other relevant clinical findings. The results from routine laboratory evaluations were normal. Radiography on the penis revealed irregular calcification in the same position as the palpable mass and in the septum of the proximal inner third of the penis (Figures 1, 2).

The calcified mass was excised surgically through a dorsal midline incision of the tunica albuginea, extending across the corpus cavernosum on both sides. The defect of the corporotomy was closed using a watertight running 4-0 vicryl suture, without grafting. A quick examination of the specimen revealed an irregular mass of grayish brown tissue with hard white calcified foci. The postoperative course was uneventful and the patient reported a full straight erection without pain. Histological examination revealed cancellous bone surrounded by dense collagen tissue.

## DISCUSSION

Several conditions have been correlated with penile ossification. The most frequent of these is Peyronie disease, but correlations with penile trauma, other diseases like metabolic disorders (for example gout and diabetes mellitus), intracavernous self-injection of vasoactive agents and chronic hemodialysis have also been reported.^3-5^ One extremely rare case of a congenital condition has been reported.^2^

McClellan was probably the first to report human penile ossification, in 1827, and Gerster and Mandelbaum were the first to do a histological study on the specimen, in 1913.^apud 2^ They concluded that the problem developed in the connective tissue, from the dorsal side of the septum between the corpora cavernosa, as a result of a metaplastic process. In 1933, Vermooten^apud2^ described a case of ossification in a man who had suffered a gunshot injury to the penis. Histopathological analysis on the mass revealed metaplastic bone marrow and cartilage formation at the fibrosis site. Numerous other cases of small calcifications in the penis have been found by macroscopic observation or X-ray. The single case of congenital ossification of the penis was described by Champion and Wegrzyn in 1964. That child also had a cleft scrotum.^2^ More recently, Vapnek reported a case of heterotopic bone formation in the corpora cavernosa of a patient with papaverine-induced priapism.^5^

It is well known that many animals present a penile bone called "os penis", "os priapi" or "baculum". It is usually located in the glans penis and aids copulation. In whales it may measure around two hundred centimeters in length and forty centimeters in circumference. In dogs it serves as a channel for the urethra, while in bears and wolves, it is essential for producing a rapid erectile state for copulation.^1^ It seems that, during later stages of evolution, the penile bone diminished in size and, in some species, appears as an insignificant structure of 10-20 mm in length. In chimpanzees, man's nearest kin, there is no "os penis", but only a virtual fragment of bone in the glans.^1^

Ossification of the cavernous tissue in humans is unrelated to phylogenetic structure. Instead of aiding copulation, as observed in the animals that have such ossification, in men it is sometimes uncomfortable and possibly painful. It is often multiple and found in the shaft as well as in the septum and tunica albuginea, while in animals it is single and situated in the glans. In most of the cases in which human penile ossification was reported, it appears to have been acquired during adult life and was related to trauma and Peyronie's disease.^1,4^ According to Devine,^6^ the fibrous tissue of the plaque can reach maturity without calcification, but calcification is a sign of the end of the healing process and may be present in 25% of the patients.

It is most likely that ossification, like the plaque in Peyronie's disease, is a scar and not the result of an inflammatory or autoimmune process. In all these conditions, human penile ossification appears to be a metaplastic process. Somers and Dawson^3^ have shown that the disease most likely begins with buckling trauma that causes injury to the septal insertion of the tunica albuginea. The fibroblastic tissue thus formed may provide good conditions for metaplastic bone formation.^4,5^

There is no good medication for treating Peyronie's disease, because few medical management methods have been subjected to doubleblind drug testing. For surgery to be considered, candidates must present mature and stable disease. It is only recommended when the curvature is enough to impair coitus.

## CONCLUSION

Our understanding of this case is that the ossification in our patient probably developed as a consequence of unusual repair of the tunica albuginea, following some blunt trauma sustained during sexual intercourse.

## References

[1] Sarma DP, Weilbaecher TG (1990). Human os penis. Urology.

[2] Champion RH, Wegrzyn J (1964). Congenital os penis. J Urol.

[3] Somers KD, Dawson DM (1997). Fibrin deposition in Peyronie's disease plaque. J Urol.

[4] Guileyardo JM, Sarma DP (1982). Human penile ossification. Urology.

[5] Vapnek J, Lue TF (1989). Heterotopic bone formation in the corpus cavernosum: a complication of papaverine-induced priapism. J Urol.

[6] Devine CJ, Horton CE (1974). Surgical treatment of Peyronie's disease with a dermal graff. J Urol.

